# No Reason to Feel Sick? Nocebo Responses in the Placebo Arms of Experimental Endotoxemia Studies

**DOI:** 10.3389/fpsyt.2019.00511

**Published:** 2019-07-17

**Authors:** Sven Benson, Sigrid Elsenbruch

**Affiliations:** Institute of Medical Psychology and Behavioral Immunobiology, University Hospital Essen, University of Duisburg-Essen, Essen, Germany

**Keywords:** nocebo response, placebo condition, immune system, inflammation, experimental endotoxemia, sickness behavior, symptom perception, side effects

## Abstract

Adverse side effects are reported by a large proportion of patients undergoing medical treatment in clinical practice or clinical trials. Nocebo effects, induced by negative treatment expectancies, can contribute to negative patient-reported outcomes but have rarely been studied in the context of inflammatory or immune-related conditions. Based on perceived treatment allocation, we herein analyzed nocebo responders in the placebo arms of randomized controlled double-blind experimental endotoxemia studies. We hypothesized that nocebo responders would report more bodily sickness symptoms and greater mood impairment. Out of *N* = 106 participants who had all received placebo injection, *N* = 20 (18.9%) wrongly believed they had received endotoxin and were thus considered as nocebo responders. Nocebo responders reported significantly more bodily sickness symptoms, suggesting that the perception of bodily symptoms affected perceived treatment allocation. Against our expectations, we did not find differences between nocebo responders and controls in psychological or physiological parameters. However, exploratory correlational analysis within nocebo responders revealed that more pronounced bodily sickness symptoms in response to placebo were associated with greater state anxiety and negative mood, as well as with the psychological traits catastrophizing and neuroticism. Our findings support that negative affectivity and personality-related factors may contribute to the reporting of sickness symptoms. Nonspecific symptoms experienced by patients undergoing pharmacological treatments or in randomized controlled trials can be misinterpreted and/or misattributed as unwanted side effects affecting perceived treatment allocation and presumably treatment satisfaction or its perceived efficacy. More nocebo research in the context of acute and chronic inflammatory conditions is warranted.

## Introduction

Adverse side effects are reported by a large proportion of patients taking medications, with negative implications for compliance, treatment continuation, and health-related quality of life (1). Owing to advances in the placebo field, it has become abundantly evident that patient-reported health outcomes including side effects are not solely explained by the specific pharmacological actions of a drug or medical treatment. Indeed, nocebo effects induced by negative treatment expectancies contribute to so-called nonspecific side effects, including the generation of unwanted side effects or the worsening of symptoms (2–4). This has been shown in the placebo arms of RCTs where the pattern of reported side effects mimics that of the verum arm (3). Nocebo effects also occur in routine care when negative treatment expectations are formed by the psychosocial treatment context, e.g., during informed consent (1, 2). Thus far, much of the existing knowledge on patient-reported nocebo effects comes from experimental pain research and the analysis of placebo arms of randomized controlled trials (RCTs). Nocebo effects have rarely been studied in the context of inflammatory or immune-related conditions, despite their broad clinical relevance (5, 6).

Aiming to close this research gap and to spark interest in translational research on nocebo effects in the context of acute inflammation, we herein analyzed nocebo-induced sickness behavior in the placebo arms of experimental endotoxemia studies. The experimental application of endotoxin is an established translational model to induce a transient systemic immune activation in healthy individuals (7). Experimental endotoxemia results in a well-characterized response encompassing psychological and bodily symptoms referred to as sickness behavior, which includes negative mood, fatigue, hyperalgesia, and nonspecific bodily symptoms (7). Sickness behavior can also occur as side effect of immune therapies and may contribute to mood disorders during chronic infection or conditions characterized by chronic inflammation (8). While many of the individual symptoms that characterize sickness behavior have been found to be modifiable by nocebo mechanisms, the collective symptom spectrum that characterizes sickness behavior in the context of acute inflammation has never been studied from a nocebo perspective.

We therefore merged data from the placebo arms of several randomized controlled double-blind endotoxemia studies conducted in our laboratory, implementing highly standardized informed consent and experimental procedures. Volunteers repeatedly received verbal and written information about effects and side effects of experimental endotoxin application during informed consent. We assessed perceived treatment allocation 24 h after the injection of placebo, assuming that an incorrect allocation (i.e., perceived endotoxin treatment when in reality received placebo) represents a nocebo responder. We compared the group of nocebo responders with volunteers with a correct treatment allocation (i.e., controls group: correct perceived allocation to placebo treatment). We specifically hypothesized that nocebo responders would report more sickness behavior symptoms, i.e., more bodily sickness symptoms and greater mood impairment. We further conducted exploratory analyses to identify psychological and physiological parameters related to the “nocebo response.”

## Material and Methods

### Participants and Study Protocol

This merged dataset comprises a total of *N* = 106 healthy volunteers (*n* = 15 women, 15.4%), who were randomized to receive a placebo injection in one of our previous (9–12) or ongoing randomized controlled double-blind endotoxemia studies. Volunteers underwent an across studies identical and highly standardized recruitment process with verbal and written information about effects and side effects of experimental endotoxin application. Rigorous screening comprising clinical and laboratory assessment was conducted at multiple time points to exclude any physical or psychological conditions. Prior participation in any experimental endotoxin study was exclusionary. Hence, participants were endotoxin-naïve herein to exclude prior experience with endotoxin-induced sickness symptoms and the study-specific psychosocial treatment context. All primary studies were conducted in medically equipped study rooms at the University Hospital Essen, Germany [for details, see Ref. (13)]. On the study days, an intravenous catheter was placed in a forearm vein for repeated blood withdrawals and for the injection of low-dose endotoxin or placebo. Before injection, volunteers were informed that they would receive either the “test substance endotoxin or an inert substance in a double-blind manner” by the study physician. Before (baseline) and up to 6 h after injection, repeated assessments (see below) of bodily and psychological sickness symptoms along with vital parameters (blood pressure, heart rate, body temperature) were conducted, and blood samples were collected for the analysis of inflammatory markers (not shown) and cortisol concentrations. Perceived treatment allocation was assessed 24 h after injection. All studies were conducted in accordance with the Declaration of Helsinki and were approved by the Institutional Ethics Review Board of the Medical Faculty of the University of Duisburg-Essen. All participants gave written informed consent and received financial compensation for study participation.

### Measures

Before the study day, psychological traits, including trait anxiety (State-Trait Anxiety Inventory, STAI-T), depression (Beck Depression Inventory, BDI), personality (NEO Five-Factor Inventory, NEO-FFI), and coping strategies (Pain-Related Self-Statement Scale, PRSS), were assessed with validated questionnaires. On the study day, state anxiety (State-Trait Anxiety Inventory, STAI-S), mood (Multidimensional Mood Questionnaire, MDBF), and bodily sickness symptoms (General Assessment of Side Effects, GASE) were repeatedly measured with standardized questionnaires. Perceived treatment allocation was retrospectively assessed 24 h after injection when volunteers returned to the lab with a brief questionnaire (forced choice of answers: believed to have received endotoxin or believed to have received placebo). Plasma cortisol concentrations were measured with commercial enzyme linked immunosorbant essay (ELISA) according to manufacturer instructions. For details on all measures, see Ref. (13).

### Statistical Analyses

Nonparametric tests were used given non-normal distribution of data. Group differences between nocebo responders and controls were analyzed with chi² and Mann–Whitney *U* tests. To test our hypotheses, nocebo responders were compared with a parallelized control group, matched for age, sex, and primary study to account for putative effects of these variables. In an additional analysis, nocebo responders were compared with the full control sample to increase transferability and transparency. To explore if specific parameters were associated with a more pronounced “nocebo response,” correlations between bodily sickness symptoms and psychological and physiological variables were computed using Spearman’s rho. If not otherwise indicated, data are shown as mean ± SD (instead of median and interquartile range) to increase clarity.

## Results

Out of *N* = 106 participants who had all received placebo injection, *N* = 20 (18.9%) wrongly believed that they had received endotoxin and were thus considered as nocebo responders. Nocebo responders did not significantly differ in sociodemographic or psychological trait variables, nor in baseline physiological (i.e., cortisol, heart rate, blood pressure, body temperature) or psychological state (i.e., state anxiety, mood) variables from parallelized and full control samples (see **Table 1**).

**Figure 1:**
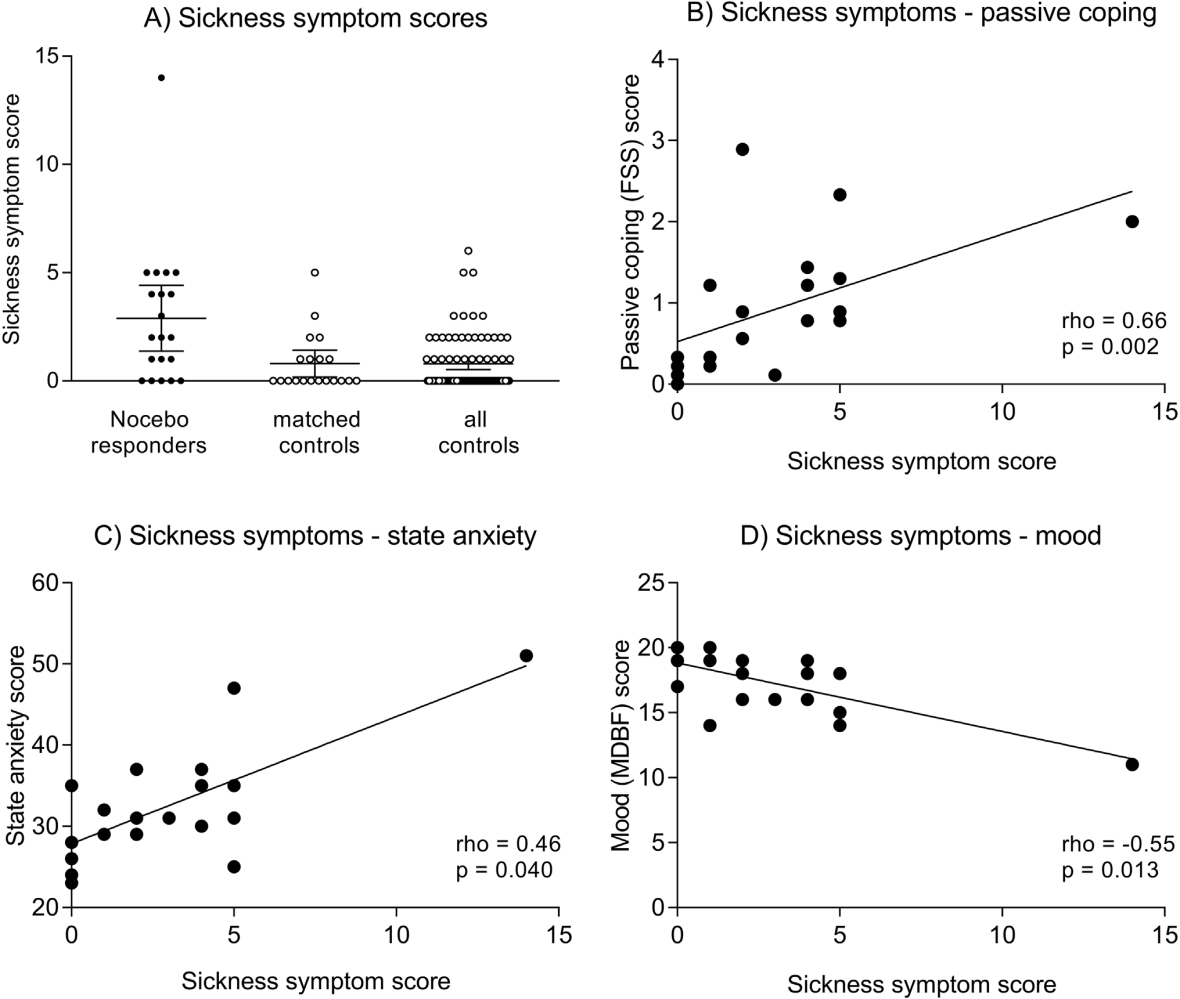
**(A)** illustrates individual sickness symptom scores in nocebo responders, matched controls, and all controls. The horizontal lines indicate mean scores and 95% CI. Group differences remain significant after exclusion of one nocebo responder with an extremely high symptom score of 14 (not shown). **Figures 1B–D** show correlations between bodily sickness symptoms and FSS passive coping scores **(B)**, State-Trait Anxiety Inventory (STAI) state anxiety scores **(C)**, and Multidimensional Mood Questionnaire, subscale negative mood. (MDBF) mood scores **(D)**. Please note that the reported correlations for mood and coping remain statistically significant after exclusion of the volunteer with a sickness symptom score of 14, while the correlation for state anxiety is no longer significant (rho = 0.37, *p* = 0.12).

In response to placebo injection, nocebo responders reported significantly more bodily sickness symptoms compared both to the parallelized (*U* = −3.12, *p* = 0.002) and full control samples (*U* = 4.05, *p* < 0.001) (**Figure 1A**, **Table 1**). Notably, differences remained significant if one nocebo responder with an extremely high symptom score of 14 was excluded from analyses (not shown). Against our expectation, we did not find evidence for increased state anxiety or impaired mood in nocebo responders (**Table 1**). In addition, no group differences were observed in blood pressure (not shown), heart rate, or plasma cortisol concentrations analyzed herein as biological markers of arousal (**Table 1**).

**Table 1:** Characteristics of nocebo responders and nonresponders.

	Nocebo responder (*N* = 20)	Matched controls (*N* = 20)^a^	Test statistic^a^	Full control sample (*N* = 86)^b^	Test statistic^b^
**Sociodemographic and psychological characteristics (trait variables)**					
Age (years)	25.9 ± 4.8	26.0 ± 4.9	*U* = −0.29, *p* = 0.78	26.8 ± 4.9	*U* = −1.16, *p* = 0.25
Sex, % (*N*)	16.0 (4)	16.0 (4)	*X*² < 0.01, *p* > 0.99	12.8 (11)	*X*² = 0.69, *p* = 0.41
Body mass index (kg/m²)	23.1 ± 2.1	23.8 ± 3.3	*U* = −0.53, *p* = 0.60	24.0 ± 2.7	*U* = −1.28, *p* = 0.20
Education > 12 years, % (*N*)	100.0 (20)	85.0 (17)	*X*² = 2.29, *p* = 0.13	89.5 (77)	*X*² = 2.29, *p* = 0.13
Trait anxiety (STAI trait)	35.9 ± 10.2	34.4 ± 7.3	*U* = −0.29, *p* = 0.78	33.4 ± 8.2	*U* = −0.81, *p* = 0.42
Depression (BDI)	3.9 ± 3.7	3.3 ± 3.2	*U* = −0.25, *p* = 0.81	3.4 ± 3.8	*U* = −0.43, *p* = 0.67
Neuroticism (NEO-FFI)	2.6 ± 1.0	2.4 ± 0.8	*U* = −0.43, *p* = 0.67	2.4 ± 0.8	*U* = −0.73, *p* = 0.47
Extraversion (NEO-FFI)	4.2 ± 0.7	4.1 ± 0.9	*U* = −0.13, *p* = 0.90	4.0 ± 0.9	*U* = −0.72, *p* = 0.48
Active coping (PRSS)	3.6 ± 0.8	3.4 ± 1.0	*U* = −0.50, *p* = 0.62	3.4 ± 1.0	*U* = −0.65, *p* = 0.52
Catastrophizing (PRSS)	0.9 ± 0.8	0.6 ± 0.6	*U* = −1.11, *p* = 0.27	0.7 ± 0.7	*U* = −1.10, *p* = 0.27
**Parameters assessed on the study day**					
Bodily sickness symptom score (GASE)	2.9 ± 3.3	0.8 ± 1.3	***U*** ** = −3.12, ** ***p*** ** = 0.002**	0.7 ± 1.2	***U*** ** = −4.05, ** ***p*** ** < 0.001**
Heart rate, baseline	67.5 ± 10.5	68.1 ± 9.8	*U* = −0.33, p = 0.74	69.9 ± 11.7	*U* = −0.74, *p* = 0.46
Heart rate, 3 h post-injection	64.4 ± 5.1	64.4 ± 8.1	*U* = −0.66, *p* = 0.51	66.6 ± 8.7	*U* = −0.72, *p* = 0.47
Heart rate, 6 h post-injection	62.8 ± 5.8	66.4 ± 7.2	*U* = −1.56, *p* = 0.14	66.9 ± 9.5	*U* = −1.83, *p* = 0.07
Plasma cortisol (nmol/l), baseline	346.9 ± 295.1	389.3 ± 252.7	*U* = −0.82, *p* = 0.43	411.7 ± 267.2	*U* = −1.30, *p* = 0.19
Plasma cortisol (nmol/l), 3 h post-injection	229.3 ± 155.8	220.1 ± 134.1	*U* = −0.01, *p* = 0.99	256.6 ± 173.9	*U* = −0.63, *p* = 0.53
Plasma cortisol (nmol/l), 6 h post-injection	197.7 ± 161.9	217.4 ± 191.5	*U* = −0.37, *p* = 0.72	238.5 ± 166.7	*U* = −1.27, *p* = 0.21
State anxiety (STAI state), baseline	34.0 ± 6.2	31.5 ± 7.0	*U* = −0.79, *p* = 0.45	34.0 ± 7.8	*U* = −0.03, *p* = 0.97
State anxiety (STAI state), 3 h post-injection	32.4 ± 7.0	31.5 ± 6.6	*U* = −0.01, *p* = 0.99	31.9 ± 6.6	*U* = −0.05, *p* = 0.96
State anxiety (STAI state), 6 h post-injection	30.1 ± 4.9	28.8 ± 5.8	*U* = −0.65, *p* = 0.52	30.3 ± 6.2	*U* = −0.23, *p* = 0.82
Negative mood (MDBF), baseline	16.9 ± 2.3	17.8 ± 1.9	*U* = −1.28, *p* = 0.20	17.5 ± 2.2	*U* = −1.38, *p* = 0.17
Negative mood (MDBF), 3 h post-injection	17.3 ± 2.4	17.6 ± 2.0	*U* = −0.19, *p* = 0.85	17.6 ± 2.2	*U* = −0.36, *p* = 0.72
Negative mood (MDBF), 6 h post-injection	18.0 ± 1.7	18.3 ± 1.6	*U* = −0.65, *p* = 0.51	18.1 ± 2.0	*U* = −0.58, *p* = 0.56

STAI, State-Trait Anxiety Inventory; BDI, Beck Depression Inventory; NEO-FFI, NEO Five-Factor-Inventory, “Big Five Personality questionnaire”; PRSS, Pain-Related Self-statement Scale; GASE, adapted version of the Global Assessment of Side Effects Scale, MDBF, Multidimensional Mood Questionnaire, subscale negative mood. Nocebo responders were compared ^a)^to a parallelized group of controls matched for age, sex, and primary study, as well as ^b)^to the full sample of controls. All data are shown as mean ± SD unless otherwise indicated. Significant group differences are printed in bold.

To explore if specific variables were associated with a more pronounced nocebo response, correlational analyses were conducted within nocebo responders. Herein, we observed that more pronounced bodily sickness symptoms were significantly correlated with PRSS catastrophizing coping (rho = 0.66, *p* = 0.002; **Figure 1B**) and with NEO-FFI neuroticism (rho = 0.49, *p* = 0.041) scores. Moreover, bodily symptoms were associated with higher state anxiety (STAI-S) assessed at 3 h postinjection (rho = 0.46, *p* = 0.040; **Figure 1C**), and with negative mood (MDBF) scores 3 h (rho = −0.55, *p* = 0.013; **Figure 1D**) and 6 h postinjection (rho = −0.46, *p* = 0.041). No significant correlations were found within the parallelized control group (all rho < 0.15, *p* > 0.56).

## Discussion

The experimental endotoxemia model offers a unique approach to analyze nocebo effects in the context of expected inflammation-induced sickness symptoms. Based on perceived treatment allocation, we herein analyzed nocebo responders within over 100 healthy volunteers in the placebo arms of randomized controlled endotoxin studies. Retrospective ratings of perceived treatment allocation revealed that ∼20% of the placebo-treated volunteers believed they had received endotoxin and were thus classified as nocebo responders. This proportion is comparable to nocebo response rates in randomized controlled drug trials, but can be even higher (14, 15). Nocebo responders reported significantly more bodily sickness symptoms, suggesting that the perception of symptoms affected perceived treatment allocation. Indeed, it has been proposed that mild, benign ailments (e.g., fatigue, headaches, drowsiness) are commonly reported even by healthy individuals not taking any medication and that such unspecific symptoms can be misattributed as unwanted drug effects in pharmacological trials (1). Supporting this notion, perceived treatment allocation was related to pain symptoms after dental surgery in clinical trials (16). Furthermore, retrospectively assessed perceived treatment allocation in a brain imaging study on placebo analgesia was preceded by alterations in neural pain processing, supporting that perceived treatment allocation is not a mere reporting bias (17). Our findings lend indirect support for the use of active placebos that mimic the (side) effects of active treatments in experimental nocebo research. If the perception of symptoms reinforces negative treatment expectations, it will indeed strengthen the assumption that an active treatment was given and hence boost nocebo effects. At the same time, active placebos could help overcome the problem of allocation concealment and blinding of patients in clinical trials (3, 18, 19).

Our second aim was to explore characteristics of nocebo responders. Against our expectations, we did not find differences between nocebo responders and controls in psychological or physiological parameters beyond bodily sickness symptom scores. However, correlational analysis revealed associations between the nocebo response and psychological parameters, which were exclusively observable within nocebo responders. This exploratory analysis suggests that nocebo responders are not characterized by alterations in psychological characteristics per se, but rather by a different contribution of psychological states and traits to the perception of sickness symptoms. In detail, we observed that more pronounced bodily sickness symptoms in response to the placebo injection were associated with greater state anxiety and negative mood, as well as with catastrophizing and neuroticism. The impact of anxiety and the anxiety-related neurotransmitter cholecystokinin on nocebo effects in pain has already been established (20). Similar processes in the perception of unspecific sickness symptoms are conceivable. It is also possible that nocebo responders misinterpreted normal somatic effects of emotional arousal induced by the injection, blood draws, or other aspects of the treatment context as side effects of endotoxin (1). Catastrophizing and neuroticism have previously been related to the perception of somatic symptoms in health and disease [e.g., Refs. (21, 22)]. Our data now support that these personality characteristics may also contribute to nocebo responses in the context of nonspecific somatic complaints. It is tempting to speculate that negative affectivity and personality-related factors have contributed to the perception and a misattribution of symptoms herein, which ultimately affected perceived treatment allocation. This would also be in line with the existing literature on predictors of nocebo responses, especially supporting a role of anxiety (23). However, current knowledge is scarce and far from conclusive (19), and our exploratory correlational findings need to be interpreted with caution. Keeping this limitation in mind, our data do not support a role of an exaggerated stress response in the generation of the nocebo response as suggested by nonsignificant findings for cortisol and heart rate. Nevertheless, future studies in animals and human should also aim to analyze the effects of repeated challenges and take the complex interaction between the generation of nocebo symptoms, aberrant neuro-immune communication, and functional changes in microglia activation (e.g., states of para-inflammation) (24) into account.

From a clinical perspective, our findings illustrate how information about immune-related sickness symptoms provided during informed consent can induce nocebo responses. Indeed, the incidence of adverse side effects after drug intake was affected by the disclosure of side effects (25–28). Another recent example is the discussion if switching from biologic agents to biosimilars may lead to nocebo responses in patients with autoimmune conditions (29). This further supports that negative information provided by health care professionals, leaflets, the media, etc. can induce nocebo effects in the context of medical interventions (2, 30), likely including those taking place in the vast clinical context of inflammation and immunity.

The strengths of our work include the translational and clinically relevant endotoxemia model with its broad spectrum of sickness symptoms, implemented using highly standardized experimental and informed-consent procedures. While this entire psychosocial treatment context invariably induces negative expectations, we unfortunately did not specifically quantify individual treatment or symptom-related expectations. This is a limitation and important future direction, as it would allow a better understanding of cognitive factors associated with nocebo responses. Furthermore, despite the large overall sample, the number of nocebo responders was small and allowed only simple correlational analyses rather than more sophisticated statistical approaches. Thus, our correlational findings do not allow causal interpretations and should be interpreted with caution. Herein, nocebo responders were classified based on a dichotomous scale. Future research could improve upon this by assessing perceived probability of a specific treatment. This would allow more refined analyses on decision making in the context of nocebo responses. It remains open if the present findings are transferrable to nocebo responses in the endotoxin arms; however, recent reports support the relevance of treatment expectations (31) and psychological parameters (13) for the intensity of sickness symptoms during real pharmacological treatment. Future research is needed to expand knowledge that herein was gathered in a small, highly selected sample of healthy young volunteers studied in an experimental laboratory setting to larger samples in clinical contexts.

## Ethics Statement

All studies were conducted in accordance with the Declaration of Helsinki and were approved by the Institutional Ethics Review Board of the Medical Faculty of the University of Duisburg-Essen. All participants gave written informed consent and received financial compensation for study participation.

## Author Contributions

SE and SB contributed conception and design of the study. SB organized the database and performed the statistical analysis. SB and SE wrote the manuscript and agree to be accountable for all aspects of the work in ensuring that questions related to the accuracy or integrity of any part of the work are appropriately investigated and resolved.

## Funding

The study was funded by the German Research Foundation (Deutsche Forschungsgemeinschaft; DFG) (to SB: BE 5173/2-1 and BE 5173/3-1; to SE: FOR 1328).

## Conflict of Interest Statement

The authors declare that the research was conducted in the absence of any commercial or financial relationships that could be construed as a potential conflict of interest.
